# Mapping of the PROMIS global health measure to the PROPr in the United States

**DOI:** 10.1186/s41687-023-00677-6

**Published:** 2024-01-10

**Authors:** Ron D. Hays, Patricia M. Herman, Nabeel Qureshi, Anthony Rodriguez, Maria Orlando Edelen

**Affiliations:** 1 Division of General Internal Medicine and Health Services Research, UCLA Department of Medicine, 1100 Glendon Avenue Suite 850, Los Angeles, CA USA; 2 RAND Corporation, Behavioral and Policy Sciences, 1776 Main Street, Santa Monica, CA USA; 3 RAND Corporation, Behavioral and Policy Sciences, 20 Park Plaza #920, Boston, MA USA; 4 Patient Reported Outcomes, Value and Experience (PROVE) Center, Department of Surgery, Brigham and Women’s Hospital, Boston, MA USA

**Keywords:** Mapping, Global physical health, Global mental health, PROPr, PROMIS®

## Abstract

**Background:**

The Patient-Reported Outcomes Measurement and Information System (PROMIS®) global health items (global-10) yield physical and mental health scale scores and the PROMIS-Preference (PROPr) scoring system estimated from PROMIS domain scores (e.g., PROMIS-29 + 2) produces a single score anchored by 0 (dead or as bad as being dead) to 1 (full health). A link between the PROMIS global-10 and the PROPr is needed.

**Methods:**

The PROMIS-29 + 2 and the PROMIS global-10 were administered to 4102 adults in the Ipsos KnowledgePanel in 2022. The median age was 52 (range 18–94), 50% were female, 70% were non-Hispanic White, and 64% were married or living with a partner. The highest level of education completed for 26% of the sample was a high school degree or general education diploma and 44% worked full-time. We estimated correlations of the PROPr with the PROMIS global health items and the global physical and mental health scales. We examined the adjusted R^2^ and estimated correlations between predicted and observed PROPr scores.

**Results:**

Product-moment correlations between the PROMIS global health items and the PROPr ranged from 0.50 to 0.63. The PROMIS global physical health and mental health scale scores correlated 0.74 and 0.60, respectively, with the PROPr. The adjusted R^2^ in the regression of the PROPr on the PROMIS global health items was 64%. The equated PROPr preference scores correlated (product-moment) 0.80 (n = 4043; p < 0.0001) with the observed PROPr preference scores, and the intra-class correlation (two-way random effects model) was 0.80. The normalized mean absolute error (NMAE) was 0.45 (SD = 0.43). The adjusted R^2^ in the OLS regression of the PROPr on the PROMIS global health scales was 59%. The equated PROPr preference scores correlated (product-moment) was 0.77 (n = 4046; p < 0.0001) with the observed PROPr preference scores, and the intra-class correlation was 0.77. The NMAE was 0.49 (SD = 0.45).

**Conclusions:**

Regression equations provide a reasonably accurate estimate of the PROPr preference-based score from the PROMIS global health items or scales for group-level comparisons. These estimates facilitate cost-effectiveness research and meta-analyses. The estimated PROPr scores are not accurate enough for individual-level applications. Future evaluations of the prediction equations are needed.

## Introduction

The Patient-Reported Outcomes Measurement Information System (PROMIS®) was developed as part of the National Institutes of Health Roadmap initiative to develop, evaluate, and standardize item banks to assess health-related quality of life across different medical conditions and in the general population [1]. The 10 PROMIS global health items (PROMIS-10) include ratings of five primary domains (physical function, fatigue, pain, emotional distress, and social health) and perceptions of general health that cut across domains. Four of the items are used to create the global physical health scale and four others to produce the global mental health scale [2]. These scales can also be estimated using two-item short forms. Internal consistency reliability coefficients for the four and two-item global physical health scales were 0.81 and 0.73, respectively, and 0.86 and 0.81 for the respective global mental health scales [3].

The PROMIS-Preference (PROPr) scoring system is based on seven PROMIS multi-item domains: physical function, pain interference, depression, fatigue, ability to participate in social roles and activities, sleep disturbance, and cognitive function. The PROMIS domain scores can be estimated from items in the domain banks, short forms (e.g., PROMIS-29 + 2), or via computer-adaptive testing [4]. All the items are administered with five polytomous response options and use a last seven-day recall period except for physical function and ability to participate in social roles and activities (which do not have an explicit recall interval).

Including a preference-based measure directly is the preferred option for obtaining a single summary score, but preference-based measures are often not administered in research studies. Many PROMIS investigators in the interest of parsimony elect to administer only the PROMIS-10 [5–7]. Being able to estimate the PROPr from the PROMIS global health items and scales provides an option for estimating a single summary score when a separate preference-based measure has not been administered.

Previous studies have used the PROMIS-10 to estimate the EQ-5D-3L [8–9] and the Health Utilities Index [10] but an estimate of the PROPr from the PROMIS-10 has not yet been published. This study derives regression equations to estimate the PROPr from the PROMIS-10.

## Methods

We administered a general health survey in English to members of KnowledgePanel®, an online panel that relies on probability-based sampling methods for recruitment and provides a representative sample of non-institutionalized adults 18 and older residing in the U.S. [11].

The survey vendor (Ipsos) sent an email invitation to 7,224 KnowledgePanel members on September 22, 2022, and gave them 10 days to complete the general health survey. Email reminders were sent to non-responders on Day 3 of the field period. Additional reminders were sent to the remaining non-responders every 3 days for up to 10 days. Upon completion, respondents received an entry into the KnowledgePanel sweepstakes. 57% (n = 4,121) completed the survey and we excluded 19 who reported having one or two of the fake health conditions [12] included in the survey to identify careless respondents, resulting in a baseline sample of 4,102.

### Measures

#### Demographic characteristics

We measured age in years, gender (female vs. male), race/ethnicity, and education: No high school diploma or general education diploma (GED); High school graduate (high school diploma or the equivalent GED); Some college or associate degree; Bachelor’s degree; Master’s degree or higher.

#### Health conditions

Thirteen health conditions were assessed by asking: “Have you ever been told by a doctor or other health professional that you had”: (1) hypertension; (2) high cholesterol; (3) heart disease; (4) angina; (5) heart attack; (6) stroke; (7) asthma; (8) cancer; (9) diabetes; (10) chronic obstructive pulmonary disease (COPD); 11) arthritis; 12) anxiety disorder; and 13) depression. In addition, the survey asked respondents if they were ever told they had “Syndomitis” (a fake condition). Further, participants were asked “Do you currently have…” 9 other conditions: (1) allergies or sinus trouble; (2) back pain; (3) sciatica; (4) neck pain; (5) trouble seeing; (6) dermatitis; (7) stomach trouble; (8) trouble hearing; and (9) trouble sleeping. Respondents were also asked if they currently had “Chekalism” (a fake condition).

#### PROMIS measures

The PROMIS global-10 includes the most widely used self-rated health item (“In general, would you say your health is…”; *global01*) and an item that provides a pure rating of physical health (global03), a rating of overall quality of life item (global02), and rating of mental health (global04). The remaining items provide global ratings of physical function (global06), fatigue (global08), pain (global07), emotional distress (global10), and social health (global05 and global09). All the items except the 0–10 rating of pain on average (global07) are administered using five-category response scales. Seven of the 10 items use a general non-specific time frame and three are prefaced with “In the past 7 days…” We scored the 10 individual PROMIS items so a higher score represents better health, and derived the global physical health (global03, global06, global07, global08) and mental health (global02, global04, global05, global10) scale scores using existing item response theory item parameters.

We used the PROPr scoring function obtained from the U.S. standard gamble valuations that yield possible scores ranging from − 0.022 to 1 [4].

#### Subjects

Those who completed the survey had a median age of 52 (range 18–94), 50% female, 70% non-Hispanic White, 64% were married or living with a spouse, the highest level of education completed for 26% of the sample was a high school degree or GED, and 44% were working full-time (Table 1). The most common health condition reported was allergies (45% of the sample), followed by hypertension and high cholesterol (38% each).

**Table 1:** Characteristic of the Sample (n = 4102)

Variable	Estimate
**Age** Median (range)	52 (18–94)
**Female**, n (%) Missing, n (%)	2032 (50%) 0 (0%)
**Race/ethnicity**, n (%)	
Hispanic	494 (12%)
Non-Hispanic	
White	2868 (70%)
Black	411 (10%)
Multi-racial	136 (3%)
Other	193 (5%)
Missing, n (%)	0 (0%)
**Marital Status**, n (%)	
Married or living with a partner	2627 (64%)
Never married	799 (19%)
Separated or divorced	460 (11%)
Widowed	216 (5%)
Missing, n (%)	0 (0%)
Education, n (%)	
Did not graduate high school	276 (7%)
High school graduate/general education diploma	1086 (26%)
Some college or Associate degree	1079 (26%)
Bachelor’s degree or higher	1661 (41%)
Missing, n (%)	0 (0%)
**Working full-time**	1822 (45%)
Missing, n (%)	10 (0.2%)
**Health conditions**	
Allergies	1857 (45%)
Missing, n (%)	8 (0.2%)
Hypertension	1569 (38%)
Missing, n (%)	1 (0.2%)
High Cholesterol	1514 (38%)
Missing, n (%)	83 (2%)
Back pain	1528 (38%)
Missing, n (%)	44 (1%)
Arthritis	1207 (30%)
Missing, n (%)	15 (0.4%)
Trouble sleeping	1143 (15%)
Missing, n (%)	9 (0.2%)
Depression	819 (20%)
Missing, n (%)	17 (0.4%)
Neck pain	807 (20%)
Missing, n (%)	7 (0.2%)
Anxiety	803 (20%)
Missing, n (%)	26 (1%)
Stomach trouble	627 (15%)
Missing, n (%)	14 (0.3%)
Sciatica	694 (17%)
Missing, n (%)	10 (0.2%)
Trouble hearing	628 (15%)
Missing, n (%)	12 (0.3%)
Trouble seeing	595 (14%)
Missing, n (%)	16 (0.4%)
Asthma	529 (13%)
Missing, n (%)	29 (1%)
Diabetes	547 (13%)
Missing, n (%)	27 (1%)
Dermatitis	421 (10%)
Missing, n (%)	17 (0.4%)
Cancer	417 (10%)
Missing, n (%)	24 (1%)
Heart disease	237 (6%)
Missing, n (%)	17 (0.4%)
Chronic obstructive pulmonary disease	191 (5%)
Missing, n (%)	29 (1%)
Myocardial infarction	120 (3%)
Missing, n (%)	25 (1%)
Stroke	106 (3%)
Missing, n (%)	40 (1%)
Angina	65 (2%)
Missing, n (%)	33 (1%)

Note: Unweighted n’s and percentages are shown. Missing percentage rows use the overall sample of 4102 as the denominator

#### Analysis plan

We estimate product-moment correlations of the PROMIS global health items and physical and mental health scale scores with the PROPr. Then, we regress the PROPr on the PROMIS global health items and then on the global physical and mental health scale scores. We used linear equating to address the problem of over-prediction of low scores and under-prediction of high scores due to regression to the mean [13]. That is, we transformed predicted scores from each of the regression models linearly to have the same mean and SD as the observed PROPr preference-based scores. We recoded scores outside of the observed range to the nearest minimum or maximum observed scores. Ordinary least squares (OLS) models were evaluated in terms of adjusted R^2^ and estimated product-moment and intraclass correlations between the predicted and observed PROPr scores. In addition, we provide Bland-Altman plots [14] with the mean of the PROPr and the equated (predicted) PROPr preference scores on the x-axis and the difference between them (PROPr – equated PROPr) on the y-axis. The 95% upper and lower limits of agreement (bias) are estimated using: mean difference +/- 1.96*SD_difference_. Scatter bias is present when the amount of disagreement between the PROPr and equated PROPr varies by the mean. We also report the normalized mean absolute error (NMAE): average deviations between observed and predicted scores divided by the standard deviation of the observed score. Lower values of the NMAE indicate better prediction. We evaluated differences in NMAE by key demographic variables: gender, age, and race/ethnicity. The magnitude of NMAE was interpreted as small or trivial for correlations of it with gender and age less than 0.243 (i.e., small correlation) and if effect size differences by race/ethnicity were less than 0.5 SD (i.e., small effect sizes or less).

## Results

### Correlations between PROMIS global health items and scale scores with the PROPr

Table 2 shows that the product-moment correlations between the PROMIS global health items and the PROPr ranged from 0.47 (global05) to 0.63 (global08). The items in the two-item global physical health short-form correlated 0.54 (global03) and 0.59 (global06) and the items in the global mental health short-form correlated 0.52 (global4) and 0.47 (global5) with the PROPr. The PROMIS four-item global physical health and mental health scale scores correlated 0.74 and 0.60, respectively, with the PROPr.

**Table 2:** Product-Moment Correlations of PROMIS Global Items with the PROPr score

PROMIS Global Health Item	PROPr Score
Global01 (overall rating of health)	0.50
Global02 (overall quality of life)	0.51
Global03 (physical health rating)	0.54
Global04 (mental health rating)	0.52
Global05 (satisfaction with social activities and relationships)	0.47
Global06 (able to carry out physical activities)	0.59
Global07 (pain on average)	0.59
Global08 (fatigue on average)	0.63
Global09 (carry out usual social activities and roles)	0.52
Global10 (bothered by emotional problems)	0.53

Note: PROMIS items should be obtained from https://www.healthmeasures.net/

### Predicting PROPr from PROMIS global health items

**Fig. 1:**
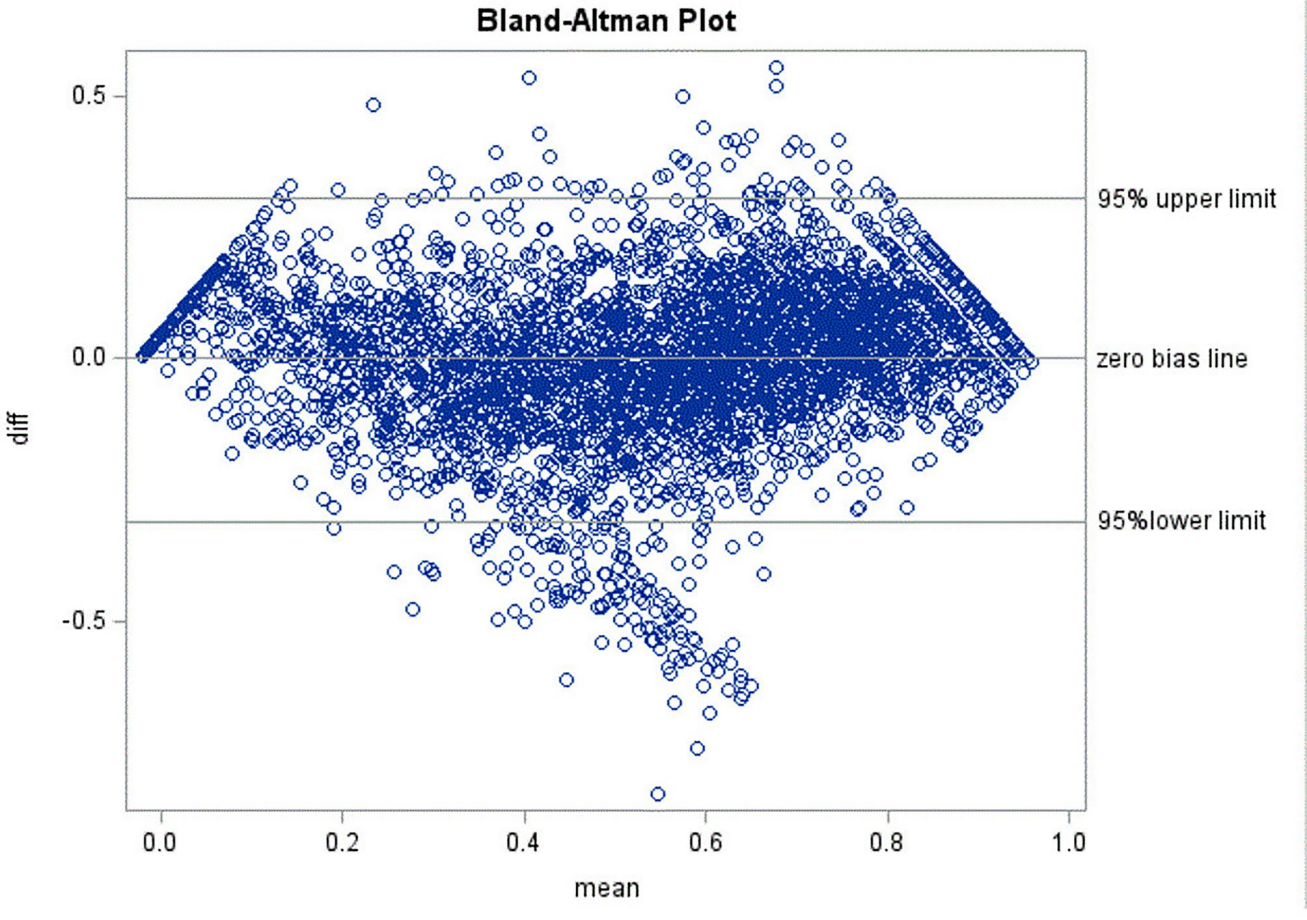
Bland-Altman Plot for Prediction of PROPr from PROMIS Global Health Items

The observed PROPr mean was 0.539 (SD = 0.249, observed score range: -0.018 to 0.954). The adjusted R^2^ in the OLS regression of the PROPr on the PROMIS global health items was 64%. The equated PROPr scores had a mean of 0.542 and an SD of 0.239. The equated PROPr preference scores correlated (product-moment) 0.80 (n=4043; p < 0.0001) with the observed PROPr preference scores, and the intra-class correlation (two-way random effects model) between observed and equated PROPr preference scores was 0.80. The NMAE was 0.45 (SD = 0.43). Although differences between predictions and PROPr scores generally were within the 95% confidence interval, the Bland-Altman plot revealed scatter bias, with the predicted values overestimating the observed PROPr scores in the middle of the distribution (Fig. 1). The equations to predict the PROPr are shown in Table 3.

**Table 3:** Equations to predict the PROPr from the PROMIS global health items and scales

**Prediction from PROMIS Global Items**: 1) PROPrpredicted = -0.54239 + 0.01548 * global03 + 0.01289 * global04 + 0.00717 * global05 + + 0.04842 * global06 + 0.01265 * global07 + 0.02568 * global08 + 0.04842 * global09 + 0.06820 * global10 2) PROPrP_equated = 0.53843+(0.24937/0.19903) * (PROPrpredicted − 0.53843) 3) if PROPrP_equated >.z and PROPrP_equated < -0.022 then PROPrP_equated = -0.022 4) If PROPrP_equated > 1 then PROPrP_equated = 1	
**Prediction from PROMIS Global Scales**: 1) PROPrpredicted = -0.68328 + 0.01820 * globalphysical + 0.00656 * globalmental 2) PROPrP_equated = 0.53847+(0.24937/0.19108) * (PROPrpredicted − 0.53847) 3) if PROPrP_equated>.z and PROPrP_equated < -0.022 then PROPrP_equated = -0.022 4) If PROPrP_equated > 1 then PROPrP_equated = 1	

The NMAE was significantly negatively correlated with age (r = -0.03, p = 0.0334) and female gender (r = -0.04, p = 0.0396), indicating that the accuracy of prediction of PROPr from the global health items was slightly lower among younger adults and males. The Tukey-Kramer multiple range test indicated that NMAE was significantly higher for non-Hispanic Blacks (mean = 0.53) and Hispanics (mean = 0.51) than for multi-racial respondents (mean = 0.43). All these differences are small in magnitude.

### Predicting PROPr from PROMIS global physical and mental health scale scores

**Fig. 2:**
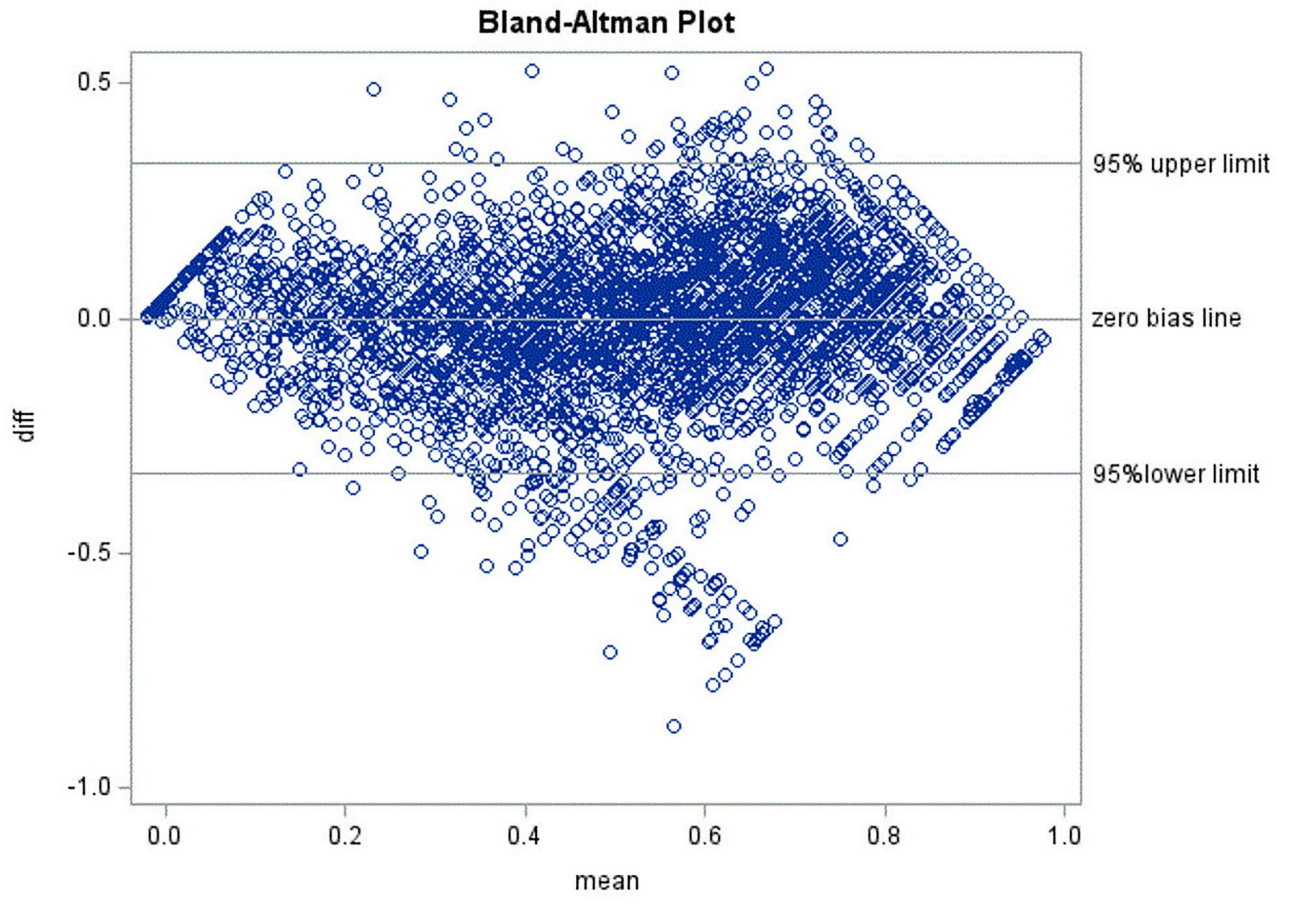
Bland-Altman Plot for Prediction of PROPr from PROMIS Global Health Scales

The adjusted R^2^ in the OLS regression of the PROPr on the PROMIS global physical and mental health scale scores was 59%. The equated PROPr scores had a mean of 0.538 and an SD of 0.238 compared with the observed PROPr mean of 0.538 and SD of 0.249. The equated PROPr preference scores correlated (product-moment) 0.77 (n = 4046; p < 0.0001) with the observed PROPr preference scores, and the intra-class correlation (two-way random effects model) between observed and equated PROPr preference scores was 0.77. The NMAE was 0.49 (SD = 0.45). The Bland-Altman plot shows scatter bias, with the predicted values overestimating the observed PROPr scores in the middle of the distribution (Fig. 2). The equations to predict the PROPr are shown in Table 3.

The NMAE was significantly negatively correlated with age (r = -0.06, p = 0.0002) and female gender (r = -0.07, p = 0.0001), indicating that the accuracy of prediction of PROPr from the global physical and mental health scale scores was slightly lower among younger adults and males. The Tukey-Kramer multiple range test indicated that the NMAE was significantly higher for non-Hispanic Blacks (mean = 0.58) and Hispanics (mean = 0.55) than for non-Hispanic Whites (mean = 0.46) and multi-racial respondents (mean = 0.43). All these differences are small in magnitude.

## Discussion

The OLS regression model indicated substantial (64% and 59%) shared variance between the PROPr and the PROMIS global health items and scale scores, respectively. The variance explained is comparable to the 65% of the variance in the EQ-5D-5L predicted by PROMIS-10 items in a previous study [8] and higher than the 40–48% of the variance shared between the PROMIS-10 and the Veterans RAND-12 physical and mental health summary scores in another study [15]. The NMAE of 0.45 (PROMIS global health items) and 0.49 (PROMIS global health scales) indicate that on average the predicted values were less than a half-standard deviation of the observed scores. Both OLS regression models were slightly less accurate in predicting PROPr scores for males, younger adults, non-Hispanic Blacks, and Hispanics.

Only two of the global health items were not significantly and uniquely related to the PROPr: the global self-rating of health (global01) and overall quality of life (global02). This is due to the previously noted [2] local dependence between global01 and the global rating of physical health (global03), and the fact that global02 is highly correlated with global ratings of mental health (global04). Both PROMIS global physical health and mental health scale scores were significantly uniquely associated with the PROPr.

While the 57% response rate exceeds the 44% average response rate found in a meta-analysis of online surveys [16], nonresponse can affect the generalizability of the results. The use of a well-known probability-based panel representative of the U.S. population [11] is a strength of the study. The unweighted sample was similar in gender and education, slightly older (52 versus 48), and had fewer Hispanics (12% versus 17%) than general population estimates from the U.S. Current Population Survey [17]. The underrepresentation of Hispanics was in part due to the limitation of the study to English-language respondents. Multivariate analyses have yielded similar results for weighted and unweighted data [18].

Methods other than OLS have been used such as Tobit and Censored Least Absolute Deviation, mixture models, and adjusted limited dependent variable mixture models to map scores from one measure to another [19]. For example, beta-binomial regression was found to perform better than OLS for several fit criteria (root mean squared error, mean absolute error, normal root mean squared error, normalized mean absolute error, and correlation between predicted and observed values) in a prior study, but the fit was similar to two decimal places (e.g., root mean squared error of 0.1218 versus 0.1191 for OLS and beta-binomial, respectively) [20]. Moreover, we used linear equating to address the problem of OLS models leading to over-predicting at the lower end and underpredicting at the upper end. Finally, the estimated scores should be limited to group-level applications because of the lack of accuracy of individual-level estimates.

Future studies are needed to further examine the accuracy of the prediction equations, but the OLS regression equations derived here can facilitate cost-effectiveness research and meta-analyses. These equations make it possible to provide a reasonable estimate of a bottom-line preference-based summary score when only the PROMIS global health items have been administered. Further exploration of the less accurate predictions for younger age, males, and among Black and Hispanic respondents is needed.

## Data Availability

The data set analyzed for this study is not publicly available yet because the project is still in progress, but the data are available from the first author on reasonable request.

## Abbreviations

PROMIS®: Patient-Reported Outcomes Measurement Information System
PROPr: PROMIS-Preference
OLS: Ordinary least squares
NMAE: Normalized mean absolute error
